# Low and High-Density Lipoprotein Cholesterol and 10-Year Mortality in Community-Dwelling Older Adults: The Shanghai Aging Study

**DOI:** 10.3389/fmed.2022.783618

**Published:** 2022-03-08

**Authors:** Wanqing Wu, Zhenxu Xiao, Xiaoniu Liang, Qianhua Zhao, Jianfeng Luo, Ding Ding

**Affiliations:** ^1^Institute of Neurology, Huashan Hospital, Fudan University, Shanghai, China; ^2^National Clinical Research Center for Aging and Medicine, Huashan Hospital, Fudan University, Shanghai, China; ^3^Department of Biostatistics, School of Public Health, Fudan University, Shanghai, China; ^4^The Key Laboratory of Public Health Safety of Ministry of Education, Shanghai, China

**Keywords:** LDL-C, HDL-C, mortality, older adults, cohort study

## Abstract

**Background:**

The relationship between serum cholesterol and mortality remains disputed. This study aimed to examine the association of low and high-density lipoprotein cholesterol (LDL-C and HDL-C) with all-cause mortality among community-dwelling older adults in the Shanghai Aging Study.

**Methods:**

We followed 3,239 participants free of lipid-lowering agents for a median of 10 years. Levels of LDL-C and HDL-C were measured at baseline using fasting blood samples. Survival status was confirmed by the local mortality surveillance system. The associations between the levels of LDL-C, HDL-C, and all-cause mortality were assessed by Cox proportional hazards models.

**Results:**

The increment of LDL-C concentration was related to a lower risk of mortality (*p* for trend < 0.05). Using the highest quintile of LDL-C (≥4.10 mmol/L) as a reference, the lowest quintile of LDL-C (<2.61 mmol/L) was associated with the highest risk of mortality, after adjusting for confounders (HR 1.67; 95% CI 1.26–2.21), exclusion of death within the first 2 years of follow-up (HR 1.57; 95% CI 1.17–2.11), and exclusion of functionally impaired participants (HR 1.46; 95% CI 1.07–2.00). A U-shape relationship was found between HDL-C level and the mortality risk. Using the third quintile of HDL-C (1.21–1.39 mmol/L) as a reference, HR (95% CI) was 1.46 (1.09–1.95) for the lowest quintile (<1.09 mmol/L) and 1.45 (1.07–1.96) for the highest quintile (≥1.61 mmol/L) of HDL-C, after adjusting for confounders; and 1.57 (1.15–2.15) for the lowest quintile and 1.45 (1.04–2.01) for the highest quintile of HDL-C, after exclusion of death within the first 2 years of follow-up; and 1.55 (1.11–2.16) for the lowest quintile and 1.42 (1.00–2.02) for the highest quintile of HDL-C, after exclusion of functionally impaired participants.

**Conclusions:**

We found an inverse association of LDL-C and a U-shape relationship of HDL-C with long-term all-cause mortality in a cohort with community-dwelling older Chinese adults. Levels of LDL-C and HDL-C are suggested to be managed properly in late life.

## Introduction

The health status of older individuals is complicated, owing to multiple subclinical and clinical diseases. Considering the complex and potentially diverse biochemical roles of cholesterol on and beyond the cardiovascular system, exploring the association between cholesterol and all-cause mortality may help to evaluate its role in late life from a comprehensive perspective.

Serum cholesterol is transported in the blood attached to lipoproteins, such as low-density lipoprotein cholesterol (LDL-C) and high-density lipoprotein cholesterol (HDL-C). The relation between late-life LDL-C, HDL-C, and all-cause mortality has been reported by population-based cohort studies. Some studies reported either a lack of an association or an inverse association for LDL-C and mortality (1–11). Some studies concluded a U-shape association for HDL-C, with both lower and higher HDL-C concentrations associated with an elevated risk of mortality (3, 12). One major methodological concern of most previous studies was lack of the adjustment of lipid-lowering therapy, a strong confounder in the association analysis. Additionally, most of the findings were from high-income countries, while large-sampled studies with long-term follow-up in the low and middle-income countries are still limited. The Shanghai Aging Study recruited a large cohort of community-dwelling older adults during 2010–2012 and prospectively monitored the survival status of the participants until the end of 2020. In this study, we aimed to examine the association between LDL-C, HDL-C level, and all-cause mortality in older Chinese adults.

## Methods

### Study Site and Population

Between 2010 and 2012, permanent residents aged 50 years or older from Jingansi community in Jingan District, Shanghai were recruited in the Shanghai Aging Study. Details of the recruiting procedure were previously published elsewhere (13). The original sample size of the Shanghai Aging Study was 3,836. In the present study, participants from the Shanghai Aging Study were excluded if they: (1) did not measure LDL-C and HDL-C at baseline (*n* = 336); (2) took lipid-lowering medication (*n* = 261). The final sample size for the current study was 3,239.

### Cholesterol Assessment

For each participant, a 2-ml fasting blood sample was drawn by a research nurse during the baseline clinical interview. Serum LDL-C and HDL-C concentration were measured by Hitachi 7 600 full-automatic biochemical analyzer with the direct method at the central lab of Huashan Hospital (13).

### Covariates

At baseline, demographic and lifestyle characteristics of the participants were collected via an interviewer-administered questionnaire including age, sex, formal educational year, family income, cigarette smoking, tea and alcohol consumption, and physical activity. Low family income was defined as per capita income <170 USD per month. Cigarette smoking status was defined if the participant had smoked daily within the past month. Alcohol consumption was defined if the participant had at least one serving of alcohol weekly during the past year. Tea consumption was defined if the participant had drunk tea more than three times a week for 6 months or over during the past year. Physically active was defined as having a total physical activity of more than 10.5 metabolic equivalent value (MET)-hours per week (14). Medical conditions such as type II diabetes, hypertension, heart diseases (including coronary heart disease, valvular heart disease, cardiomyopathy, heart failure, heart rhythm problems), stroke, and cancer were asked and further confirmed from the medical records. The Center for Epidemiologic Studies Depression Scale (CESD) was administered to assess psychiatric status. Depression was present if CESD was ≥ 16 (13). Activities of Daily Living (ADL) were used for evaluating the functional ability. Participants were defined as functionally impaired if ADL was >20 (15). Anthropometry was performed by research nurses. Height and weight were used to calculate the body mass index (BMI). Obesity was defined as BMI ≥27.5 kg/m^2^ based on the World Health Organization (WHO)'s definition for Asian populations (16).

### Mortality Surveillance

The survival status of participants from baseline to December 31, 2020, was confirmed by access to the mortality surveillance system in the Center of Disease Control (CDC) in Jingan District, Shanghai. According to the regulations on household registration of Shanghai, once the death of the resident occurs, the CDC in his/her registered permanent residence is responsible for verifying the date of death and fundamental cause of death, which is coded by the International Classification of Diseases, tenth edition (ICD-10) from the death certificate.

### Statistical Analysis

Mean with standard deviation (SD) and number with frequency (%) were used to describe continuous and categorical variables, respectively. Participants were categorized into subgroups according to the quintiles of LDL-C and HDL-C concentration. *T*-test was used to analyze the differences for continuous variables and Pearson's chi-squared test was used for categorical variables, between participants who took lipid-lowering medications and those who did not.

Participants were followed up for their survival status from baseline (2010–2012) to December 31, 2020. For those deceased, survival time was defined as the difference between the date of death and the date of baseline when levels of LDL-C and HDL-C were measured. Participants were censored as long as they were alive until December 31, 2020. The follow-up length was then defined as the difference between the date of baseline examination and the date of December 31, 2020. The crude mortality rate was calculated as the number of deaths divided by the cumulative person-years of follow-up. The hazard ratios (HRs) of mortality for LDL-C and HDL-C quintiles were estimated using Cox proportional hazards models. Model 1 adjusted for age, sex, years of formal education, low family income, cigarette smoking, tea and alcohol consumption, physical activity, and obesity. Model 2 further adjusted for a history of type II diabetes, hypertension, heart diseases, stroke, cancer, and depression. Model 3 excluded those who died within the first 2 years of follow-up based on model 2. Model 4 excluded those who were functionally impaired based on model 2. The linear trend was tested by entering LDL-C and HDL-C as continuous variables. Adjusted cumulative survival curves were plotted based on the results of model 2. Subgroup analysis was also conducted according to the median age and sex, based on model 2. We have tested the proportional hazards (PH) assumption using Schoenfeld residuals methods and Log-log survival curves. The result of Schoenfeld residuals methods and the curves of Log-log survival analysis suggested that the PH assumption was not violated for all variables included in the Cox models.

All the *p*-values and 95% CIs were estimated in two-tailed tests. The data analysis was conducted using SAS 9.4 (SAS Institute Inc., Cary, NC, USA).

## Results

Comparison of the baseline characteristics between participants who took lipid-lowering medications (*n* = 261) and those who did not (*n* = 3,239) was shown in Supplementary Table. In general, participants who took medications were older and had a higher prevalence of chronic diseases such as hypertension, diabetes, stroke, and heart diseases, compared to those who did not. Also, those who took medications had a significantly lower level of LDL-C and HDL-C.

In total, 3,239 participants were included in this study. The baseline characteristics of the study participants were shown in Table 1. Participants' mean age was 69.41 ± 8.05 years old and the average year of education was 11.67 ± 4.02 years. Half of the participants had hypertension, and the prevalence of other medical conditions was about 10%. The means of LDL-C and HDL-C concentrations were 3.35 ± 0.91 mmol/L and 1.35 ± 0.35 mmol/L. Participants with higher LDL-C were younger, more likely to be female, had a higher level of BMI, had a lower prevalence of type II diabetes, and had a higher level of HDL-C.

**Table 1:** Baseline characteristics of study participants.

	**Total**	**Quintiles of LDL-C**				
		** <2.61 mmol/L**	**2.61–3.09 mmol/L**	**3.10–3.50 mmol/L**	**3.51–4.09 mmol/L**	**≥4.10 mmol/L**
Age, years, mean (SD)	69.41 (8.05)	70.18 (8.01)	69.84 (8.13)	69.55 (7.95)	69.24 (8.40)	68.31 (7.68)
Male, *n* (%)	1,476 (45.57)	362 (54.77)	302 (50.25)	303 (44.23)	268 (43.37)	241 (35.76)
Education, years, mean (SD)	11.67 (4.02)	11.64 (4.13)	11.39 (4.15)	11.75 (3.99)	11.73 (4.01)	11.80 (3.82)
BMI, mean (SD)	24.30 (3.45)	23.99 (3.59)	24.05 (3.53)	24.17 (3.42)	24.50 (3.42)	24.76 (3.22)
Low family income, *n* (%)	60 (1.86)	9 (1.37)	17 (2.83)	8 (1.18)	13 (2.11)	13 (1.94)
Cigarette smoking, *n* (%)	356 (10.99)	76 (11.50)	63 (10.48)	80 (11.68)	63 (10.19)	74 (10.98)
Alcohol consumption, *n* (%)	271 (8.37)	53 (8.02)	38 (6.32)	72 (10.51)	54 (8.74)	54 (8.01)
Tea drinking, *n* (%)	1,381 (42.93)	282 (42.86)	246 (41.07)	303 (44.69)	262 (42.74)	288 (43.05)
Physically active, *n* (%)	2,003 (62.28)	393 (60.00)	383 (64.05)	440 (64.99)	383 (62.18)	404 (60.30)
Obesity, *n* (%)	545 (16.87)	108 (16.34)	95 (15.86)	107 (15.64)	105 (17.07)	130 (19.37)
Hypertension, *n* (%)	1,601 (49.43)	334 (50.53)	309 (51.41)	328 (47.88)	316 (51.13)	314 (46.59)
Type II diabetes, *n* (%)	408 (12.60)	118 (17.85)	72 (11.98)	85 (12.41)	69 (11.17)	64 (9.50)
Stroke, *n* (%)	351 (10.83)	73 (11.04)	72 (11.98)	72 (10.51)	68 (11.00)	66 (9.79)
Heart diseases, *n* (%)	300 (9.26)	65 (9.83)	47 (7.82)	72 (10.51)	60 (9.71)	56 (8.31)
Cancer, *n* (%)	318 (9.87)	78 (11.85)	60 (10.03)	63 (9.24)	69 (11.24)	48 (7.15)
Depression, *n* (%)	557 (17.20)	116 (17.55)	104 (17.30)	117 (17.08)	101 (16.34)	119 (17.66)
LDL-C, mmol/L, mean (SD)	3.35 (0.91)	2.15 (0.40)	2.87 (0.12)	3.29 (0.13)	3.78 (0.15)	4.64 (0.55)
HDL-C, mmol/L, mean (SD)	1.35 (0.35)	1.27 (0.40)	1.32 (0.34)	1.35 (0.33)	1.36 (0.33)	1.43 (0.32)

*BMI, body mass index; LDL-C, low-density lipoprotein cholesterol; HDL-C, high-density lipoprotein cholesterol*.

**Figure 1:**
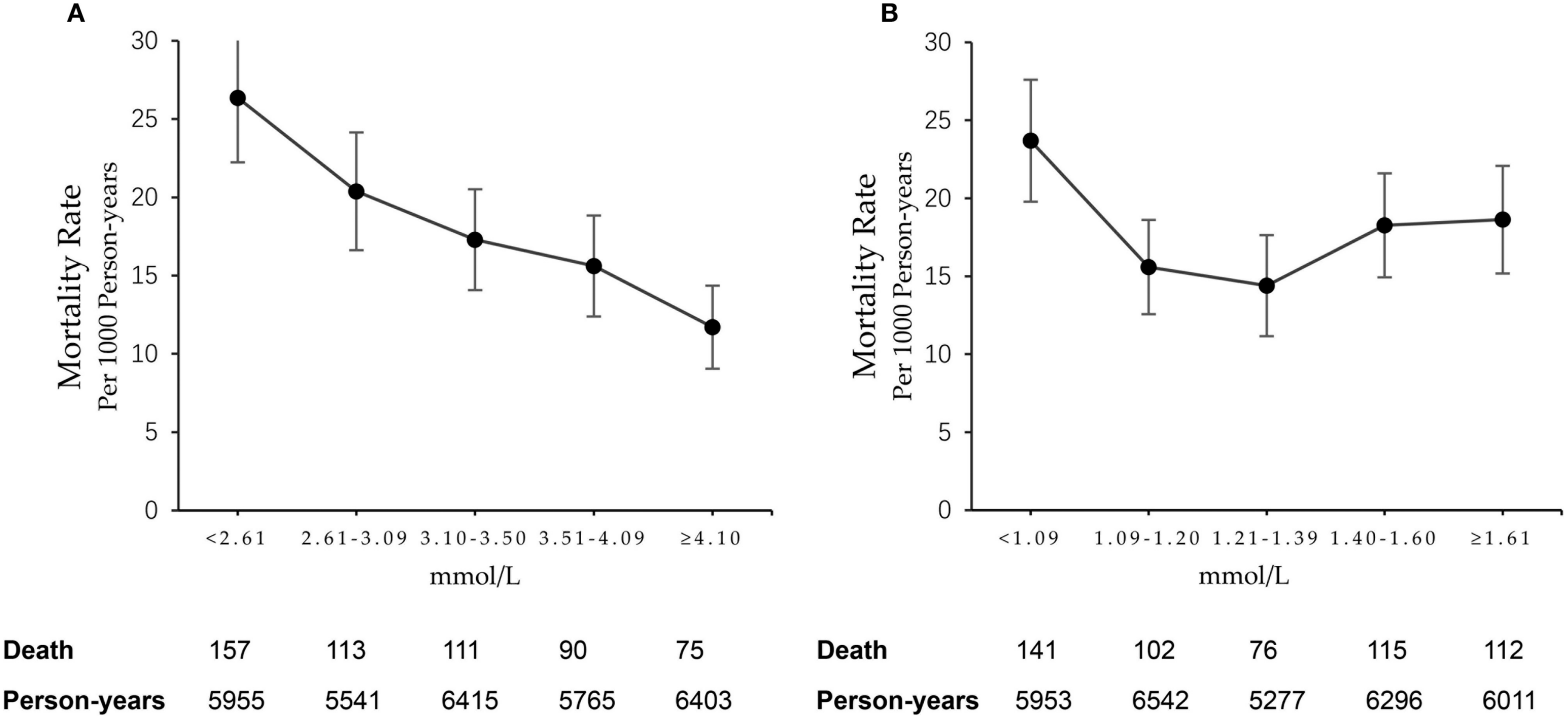
Mortality rates in subgroups according to quintiles of **(A)** LDL-C and **(B)** HDL-C.

Five hundred and forty-six deaths (16.9%) were identified during a median of 10-year follow-up. Figure 1A revealed a monotonic decrease of the crude mortality rate with the increment of LDL-C concentration, from 26.36 (95% CI: 22.24–30.49) per 1 000 person-years in the lowest quintile to 11.71 (95% CI: 9.06–14.36) per 1 000 person-years in the highest quintile. Figure 1B presented a curvilinear trend between HDL-C and mortality, with the lowest mortality rate of 14.40 (95% CI: 11.16–17.64) per 1 000 person-years in the group of HDL-C concentration of 1.21–1.39 mmol/L.

As shown in Table 2, the increment of LDL-C concentration was related to decreasing risk of mortality (*p* for trend < 0.05). Using the highest quintile of LDL-C (≥4.10 mmol/L) as a reference, the lowest quintile (<2.61 mmol/L) was associated with the highest risk of mortality, after adjusting for confounders (HR 1.67; 95% CI 1.26–2.21), exclusion of death within the first two years of follow-up (HR 1.57; 95% CI 1.17–2.11), and exclusion of functionally impaired participants (HR 1.46; 95% CI 1.07–2.00).

**Table 2:** Adjusted hazard ratio (95% confidence interval) of all-cause mortality.

	**Model 1**	***p* for trend**	**Model 2**	***p* for trend**	**Model 3**	***p* for trend**	**Model 4**	***p* for trend**
**LDL-C**		<0.001		<0.001		<0.001		0.011
<2.61 mmol/L	1.70 (1.28–2.25)*		1.67 (1.26–2.21)*		1.57 (1.17–2.11)*		1.46 (1.07–2.00)*	
2.61–3.09 mmol/L	1.30 (0.97–1.75)		1.28 (0.95–1.73)		1.20 (0.88–1.64)		1.17 (0.84–1.62)	
3.10–3.50 mmol/L	1.20 (0.89–1.61)		1.14 (0.85–1.54)		1.18 (0.87–1.61)		1.12 (0.81–1.55)	
3.51–4.09 mmol/L	1.05 (0.77–1.43)		1.02 (0.75–1.39)		0.96 (0.69–1.33)		0.95 (0.67–1.35)	
≥4.10 mmol/L	1		1		1		1	
**HDL-C**		0.732		0.878		0.494		0.426
<1.09 mmol/L	1.48 (1.11–1.98)*		1.46 (1.09–1.95)*		1.57 (1.15–2.15)*		1.55 (1.11–2.16)*	
1.09–1.20 mmol/L	1.04 (0.77–1.41)		1.05 (0.77–1.42)		1.11 (0.81–1.54)		1.19 (0.84–1.67)	
1.21–1.39 mmol/L	1		1		1		1	
1.40–1.60 mmol/L	1.20 (0.89–1.61)		1.21 (0.89–1.63)		1.27 (0.92–1.74)		1.37 (0.98–1.92)	
≥1.61 mmol/L	1.41 (1.05–1.91)*		1.45 (1.07–1.96)*		1.45 (1.04–2.01)*		1.42 (1.00–2.02)*	

*Model 1 adjusted for age, sex, educational years, low family income, cigarette smoking, alcohol drinking, tea-drinking, physical activity, and obesity*.

*Model 2 further adjusted for hypertension, type II diabetes, stroke, heart diseases, cancer, and depression*.

*Model 3 excluded those who died within 2 years of initial cholesterol measurement (n = 60) on the basis of model 2*.

*Model 4 excluded those who were functionally impaired (n = 212) on the basis of model 2*.

*LDL-C, low-density lipoprotein cholesterol; HDL-C, high-density lipoprotein cholesterol*.

* *p < 0.05*.

A U-shape relationship was found between HDL-C level and mortality risk, with both the lowest and highest quintile of HDL-C being associated with increased mortality risk. Using the third quintile of HDL-C (1.21–1.39 mmol/L) as a reference, HR (95% CI) was 1.46 (1.09–1.95) for the lowest quintile (<1.09 mmol/L) and 1.45 (1.07–1.96) for the highest quintile (≥1.61 mmol/L) of HDL-C, after adjusting for confounders; and 1.57 (1.15–2.15) for the lowest quintile and 1.45 (1.04–2.01) for the highest quintile of HDL-C, after exclusion of death within the first 2 years of follow-up; and 1.55 (1.11–2.16) for the lowest quintile and 1.42 (1.00–2.02) for the highest quintile of HDL-C, after exclusion of functionally impaired participants.

**Figure 2:**
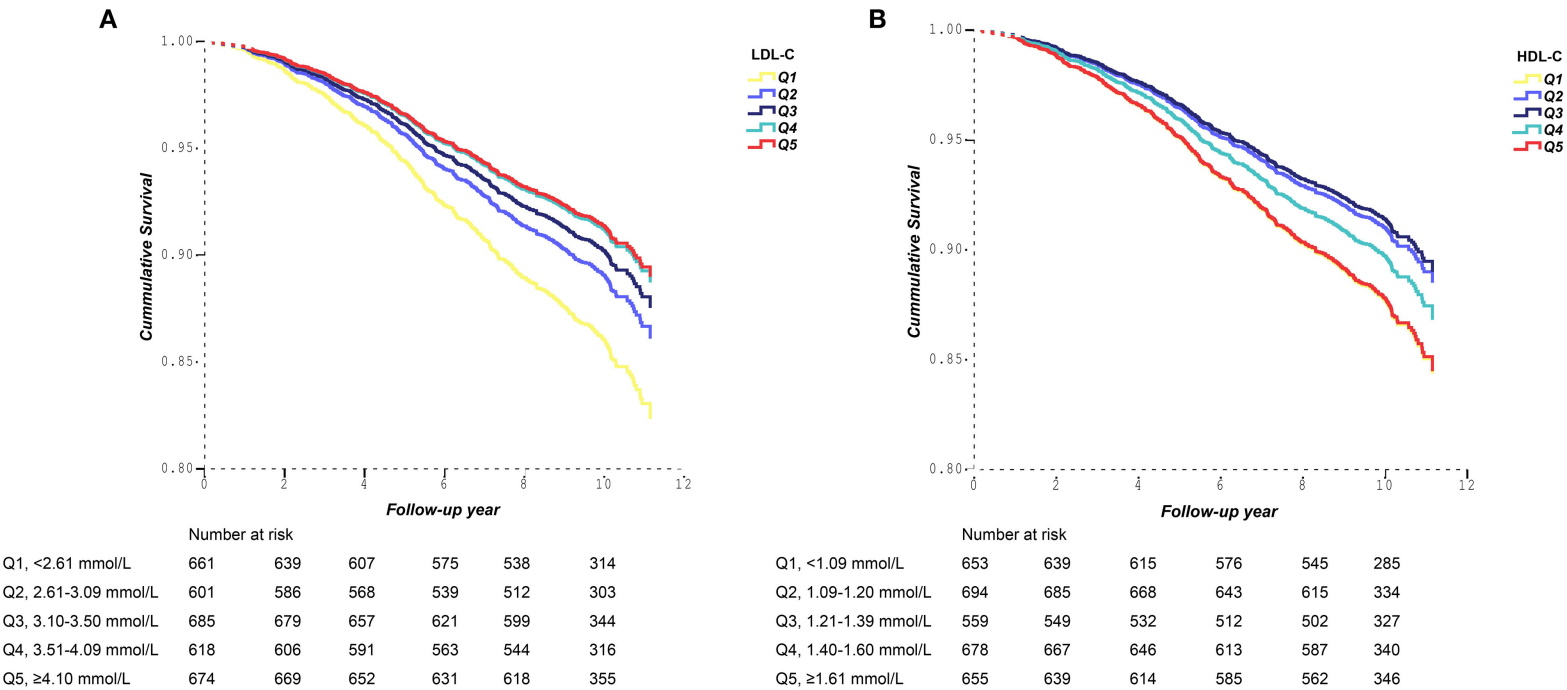
Survival curves in subgroups according to quintiles of **(A)** LDL-C and **(B)** HDL-C.

Figure 2 demonstrated the cumulative survival curves of participants according to the quintiles of LDL-C and HDL-C levels. Participants with the lowest LDL-C concentration (Q1) showed the worst survival, followed by those in the second and third quintile (Q2, Q3). Participants with LDL-C in the fourth and fifth quintiles (Q4, Q5) shared the best cumulative survival. The difference in the cumulative survival between Q1, Q2 and Q3, Q4 and Q5 of LDL-C expanded over time (Figure 2A). The best survival was found for participants in the third HDL-C quintile. Both the lowest and the highest HDL-C quintiles presented a worse survival compared to the third HDL-C quintile (Figure 2B).

The results of the subgroup analysis were shown in Table 3. The inverse association between LDL-C and 10-year all-cause mortality risk was observed among participants ≥68 years old (*p* < 0.001), both females (*p* < 0.001) and males (*p* = 0.016). The U-shape relationship in which both the lowest and highest quintile of HDL-C were related to increased mortality risk was observed among participants ≥68 years old (HR 1.60; 95% CI 1.18–2.19 for the lowest quintile, HR 1.64; 95% CI 1.19–2.25 for the highest quintile) and males (HR 1.57; 95% CI 1.08–2.29 for the lowest quintile, HR 1.72; 95% CI 1.21–2.26 for the highest quintile). For females, only the lowest quintile of HDL-C was related to increased mortality risk (HR 1.94; 95% CI 1.27–2.96).

**Table 3:** Adjusted hazard ratio (95% confidence interval) of all-cause mortality among the subgroups.

**Age <68 years**		**Age ≥68 years**		**Female**		**Male**	
**LDL-C**							
<2.70 mmol/L	1.76 (0.94, 3.31)	<2.60 mmol/L	1.76 (1.28, 2.43)*	<2.71 mmol/L	2.29 (1.47, 3.57)*	<2.50 mmol/L	1.58 (1.10, 2.28)*
2.71–3.14 mmol/L	1.19 (0.61, 2.31)	2.61–3.01 mmol/L	1.27 (0.91, 1.76)	2.71–3.20 mmol/L	1.24 (0.77, 1.99)	2.50–2.98 mmol/L	1.08 (0.73, 1.59)
3.15–3.60 mmol/L	1.13 (0.58, 2.22)	3.02–3.45 mmol/L	1.27 (0.91, 1.77)	3.21–3.64 mmol/L	1.19 (0.72, 1.97)	2.99–3.39 mmol/L	1.31 (0.89, 1.92)
3.61–4.16 mmol/L	0.79 (0.37, 1.70)	3.46–4.00 mmol/L	1.06 (0.75, 1.49)	3.65–4.20 mmol/L	1.21 (0.73, 1.98)	3.40–3.90 mmol/L	1.08 (0.74, 1.59)
≥4.17 mmol/L	1	≥4.01 mmol/L	1	≥4.21 mmol/L	1	≥3.91 mmol/L	1
*p* for trend	0.104	*p* for trend	<0.001	*p* for trend	<0.001	*p* for trend	0.016
**HDL-C**							
<1.09 mmol/L	1.14 (0.62, 2.10)	<1.09 mmol/L	1.60 (1.18, 2.19)*	<1.10 mmol/L	1.94 (1.27, 2.96)*	<1.00 mmol/L	1.57 (1.08, 2.29)*
1.09–1.20 mmol/L	0.90 (0.47, 1.72)	1.09–1.20 mmol/L	1.14 (0.83, 1.57)	1.10–1.29 mmol/L	0.95 (0.62, 1.45)	1.00–1.11 mmol/L	1.18 (0.81, 1.73)
1.21–1.39 mmol/L	1	1.21–1.40 mmol/L	1	1.30–1.49 mmol/L	1	1.12–1.29 mmol/L	1
1.40–1.61 mmol/L	1.14 (0.60, 2.17)	1.41–1.60 mmol/L	1.39 (1.00, 1.93)	1.50–1.70 mmol/L	0.94 (0.61, 1.46)	1.30–1.49 mmol/L	1.15 (0.78, 1.68)
≥1.62 mmol/L	0.87 (0.43, 1.76)	≥1.61 mmol/L	1.64 (1.19, 2.25)*	≥1.71 mmol/L	1.20 (0.78, 1.86)	≥1.50 mmol/L	1.72 (1.21, 2.46)*
*p* for trend	0.239	*p* for trend	0.855	*p* for trend	0.088	*p* for trend	0.155

*Hazard ratio (95% confidence interval) were adjusted for age, sex, educational years, low family income, cigarette smoking, alcohol drinking, tea-drinking, physical activity, obesity, hypertension, type II diabetes, stroke, heart diseases, cancer, and depression. LDL-C, low-density lipoprotein cholesterol; HDL-C, high-density lipoprotein cholesterol*.

* *p < 0.05*.

## Discussion

In this population-based prospective study, we observed an inverse association between LDL-C and 10-year all-cause mortality risk among 3,239 older adults without using lipid-lowering agents. The relationship of HDL-C with all-cause mortality demonstrated a U-shape, both the lowest and highest quintile of HDL-C were related to increased mortality risk.

Evidence was limited regarding the association of late-life LDL-C, HDL-C, and all-cause mortality in low and middle-income countries. Our study was conducted in a large sample of Chinese community-dwellers through a long-term follow-up. The information on the participants' survival status in our study was from the mortality surveillance system in the local CDC with a follow-up rate of 100%. We excluded older adults who were on lipid-lowering medication, a potentially strong confounder for lipids and mortality. Additionally, we excluded participants who died within the first 2 years of follow-up and those with functional impairment to prevent reverse causality.

Our finding is consistent with previous studies where an inverse association of LDL-C with all-cause mortality was found among older adults (2–7). Using a large sample size (*N* = 13,733), a population-based register study in Region Zealand reported an increased risk of death for older adults with LDL-C <2.5 mmol/L (3). Another observational study in Italy found that the risk of death was reduced by 16% for every 1 mmol/L of LDL-C increment (2). Similar inverse associations of LDL-C with risk of death were also reported in other population-based observational studies from the USA, Finland, Netherland, Japan, and China (1, 4–7). Several studies reported no significant association between LDL-C and all-cause mortality (8–11). For example, the Cardiovascular Health Study found LDL-C was inversely associated with mortality in the crude model, but not in the model adjusting for potential confounders (8). Although LDL-C is a well-established risk factor for atherosclerotic cardiovascular disease and results from the Cooper Center Longitudinal Study showed that high levels of LDL-C were independently associated with an increased risk of cardiovascular disease mortality (17), to our knowledge, no studies reported a positive association between late-life LDL-C and all-cause mortality. Combined, these studies indicate that high late-life LDL-C levels do not seem to be definitely harmful in the general population. High LDL-C might not be a risk indicator at old age and thus be used to identify older adults at risk and start cardiovascular disease management. Instead, those with low LDL-C in late life might warrant further attention. An adjustment of diet may help to increase their level of LDL-C and avoid extra risk of all-cause mortality.

Our findings could be explained by that among older individuals, lower LDL-C levels are partially a surrogate marker of frailty. Evidence shows that LDL-C gradually decreases in the latter decades of life (18). The ability to preserve a higher level of LDL-C in late life may represent a better global health condition and protect individuals from death. Large-scale epidemiologic studies, such as the Whitehall Study, the Framingham Study, and the Honolulu Study have related low cholesterol to a higher cancer incidence and mortality, suggesting a potential role of cholesterol in cancer development and survival (19). Higher serum cholesterol also promotes inflammatory responses, including augmentation of toll-like receptor signaling, inflammasome activation, and the production of monocytes and neutrophils in the bone marrow and spleen (20).

Our finding of a U-shape relationship between HDL-C and all-cause mortality is consistent with the findings of the Health and Retirement Study (HRS) and the population-based register study in Region Zealand (3, 12). Both low and high HDL-C were associated with an increased risk of death. In HRS, the risk of death was increased by 72% and 56% for participants with HDL-C <30 mg/dl and HDL-C ≥ 90 mg/dl, respectively, compared with those with HDL-C of 70–79 mg/dl (12). Other studies reported either no association or only low HDL-C was associated with high all-cause mortality (1, 4, 6, 11, 21). In a prospective cohort in Finland, older adults in the lowest quartile of HDL-C were over twice as likely to die as those in the highest quartile (1). Although the diverse results and potential mechanisms could not be well explained, these findings add to the uncertainty of the role of HDL-C for mortality in old people.

Subgroup analysis showed a similar association between LDL-C/HDL-C and 10-year all-cause mortality risk among participants ≥68 years old, both females and males. Although we did not observe an inverse association of LDL-C with all-cause mortality among participants <68 years, the risk estimates for the lowest quintile of LDL-C were similar between those younger than 68 and those ≥68. Thus, a limited number of deaths among participants <68 years may account for the insignificant finding. For females, although the lowest quintile of HDL-C was related to increased mortality risk, the increased risk for the highest quintile of HDL-C did not reach a statistical significance. The effect of a higher level of HDL-C on mortality might be varied across sex.

The limitations of our study are listed as the following. First, the current study relied on a single measurement of LDL-C and HDL-C at baseline. Measurement error and biological variability would impact the association estimation. Second, reverse causality may be of concern due to the potential underlying chronic diseases of older adults. However, in our study, the inverse association remained unchanged after excluding those who died in the first 2 years of follow-up and those who were functionally impaired. Rather, the difference of cumulative survival between high and low LDL-C levels expanded over time, which strengthens the evidence against reverse causality. Third, despite adjustment for important covariates in our analysis, the possibility of residual confounding from unmeasured variables such as diets, occupation history, and air pollution could also affect the results. Fourth, our study only focused on all-cause mortality because the relatively small number of events has limited the ability to study cause-specific mortality. In addition, the current analysis is a *post-hoc* analysis. The sample size was not calculated according to mortality rate. Last, our study participants were recruited from communities in downtown Shanghai using a government-maintained “residents list”. The population-based nature of the Shanghai Aging Study indicates that the study sample well-represented the characteristics of older adults living in downtown Shanghai. However, our findings may not be generalized to other populations in China in which the demographic characteristics could be varied.

In conclusion, we found an inverse association of LDL-C and a U-shape relation of HDL-C with long-term all-cause mortality in a cohort with community-dwelling older Chinese adults. Levels of LDL-C and HDL-C are suggested to be managed properly in late life. The role of LDL-C and HDL-C in health status in late life needs further validation in diverse regions and ethnic populations. The causal relation cannot be answered with the observational design, whereas the randomized clinical trials so far indicate that treatment with cholesterol-lowering drugs is of benefit also in older age. The decision whether or not to treat older people with cholesterol-lowering drugs needs to weigh the “costs” and the “benefits” for the individual and society.

## Data Availability Statement

The original contributions presented in the study are included in the article/Supplementary Material, further inquiries can be directed to the corresponding author.

## Ethics Statement

The studies involving human participants were reviewed and approved by the Medical Ethics Committee of Huashan Hospital, Fudan University, Shanghai, China. The patients/participants provided their written informed consent to participate in this study.

## Author Contributions

DD was responsible for the study's concept and design. WW, DD, ZX, XL, and QZ collected the data. WW did the analyses with support from JL. WW prepared the manuscript. DD, XL, ZX, QZ, and JL revised the manuscript. DD is the guarantor of this article. All authors contributed to the article and approved the submitted version.

## Funding

This work was funded by grants from Scientific Research Plan Project of Shanghai Science and Technology Committee [17411950701 and 17411950106], National Natural Science Foundation of China [81773513], Shanghai Municipal Science and Technology Major Project [No.2018SHZDZX01] and ZJ Lab, and National Project of Chronic Disease [2016YFC1306402]. Funders had no role in the design, execution, analysis, and interpretation of data, or writing of the study.

## Conflict of Interest

The authors declare that the research was conducted in the absence of any commercial or financial relationships that could be construed as a potential conflict of interest.

## Publisher's Note

All claims expressed in this article are solely those of the authors and do not necessarily represent those of their affiliated organizations, or those of the publisher, the editors and the reviewers. Any product that may be evaluated in this article, or claim that may be made by its manufacturer, is not guaranteed or endorsed by the publisher.
