# Supplementary material for: Low and High-Density Lipoprotein Cholesterol and 10-Year Mortality in Community-Dwelling Older Adults: The Shanghai Aging Study
Source: Front Med (Lausanne). 2022 Mar 8;9:783618. doi: 10.3389/fmed.2022.783618 (PMC8959128; doi:10.3389/fmed.2022.783618)
Supplement: Supplementary file 1 [file Table_1.pdf]

Supplementary Table. Comparision of baseline characteristics according to whether taking cholesterol-lowering medication or not

	Not taking cholesterol-lowering medication (n=3239)	Taking cholesterol-lowering medication (n=261)	*p-value*
Age, years, mean (SD)	69.41 (8.05)	71.08 (8.01)	0.001
Male, n (%)	1476 (45.57)	116 (44.44)	0.726
Education, years, mean (SD)	11.67 (4.02)	12.15 (4.54)	0.096
BMI, mean (SD)	24.30 (3.45)	24.83 (3.32)	0.015
Low family income, n (%)	60 (1.86)	5 (1.92)	0.944
Cigarette smoking, n (%)	356 (10.99)	22 (8.43)	0.200
Alcohol consumption, n (%)	271 (8.37)	14 (5.36)	0.088
Tea drinking, n (%)	1381 (42.93)	120 (46.69)	0.241
Physically active, n (%)	2003 (62.28)	144 (55.81)	0.040
Obesity, n (%)	545 (16.87)	46 (17.62)	0.756
Hypertension, n (%)	1601 (49.43)	187 (71.65)	<0.001
Type II diabetes, n (%)	408 (12.60)	56 (21.46)	<0.001
Stroke, n (%)	351 (10.83)	56 (21.46)	<0.001
Heart diseases, n (%)	300 (9.26)	83 (31.80)	<0.001
Cancer, n (%)	318 (9.87)	30 (11.49)	0.399
Depression, n (%)	557 (17.20)	49 (18.77)	0.517
LDL-C, mmol/L, mean (SD)	3.35 (0.91)	3.00 (0.98)	<0.001
HDL-C, mmol/L, mean (SD)	1.35 (0.35)	1.30 (0.32)	0.049

BMI, body mass index; LDL-C, low-density lipoprotein cholesterol; HDL-C, high-density lipoprotein cholesterol.
